# Insights into the Structural, Thermal/Dilatometric, and Optical Properties of Dy^3+^-Doped Phosphate Glasses for Lighting Applications

**DOI:** 10.1021/acsphyschemau.4c00066

**Published:** 2024-10-21

**Authors:** José A. Jiménez, Vinod Hedge, C. S. Dwaraka Viswanath, Richard Amesimenu

**Affiliations:** † Center for Advanced Materials Science, Department of Biochemistry, Chemistry & Physics, Georgia Southern University, Statesboro, Georgia 30460, United States; ‡ Department of Physics, Manipal Institute of Technology Bengaluru, Manipal Academy of Higher Education, Manipal, Karnataka 576104, India; § Department of Science and Humanities, Mother Theresa Institute of Engineering and Technology, Palamaner, Andhra Pradesh 517408, India

**Keywords:** phosphate glasses, lanthanides, optical properties, spectroscopy, structural properties, thermal properties

## Abstract

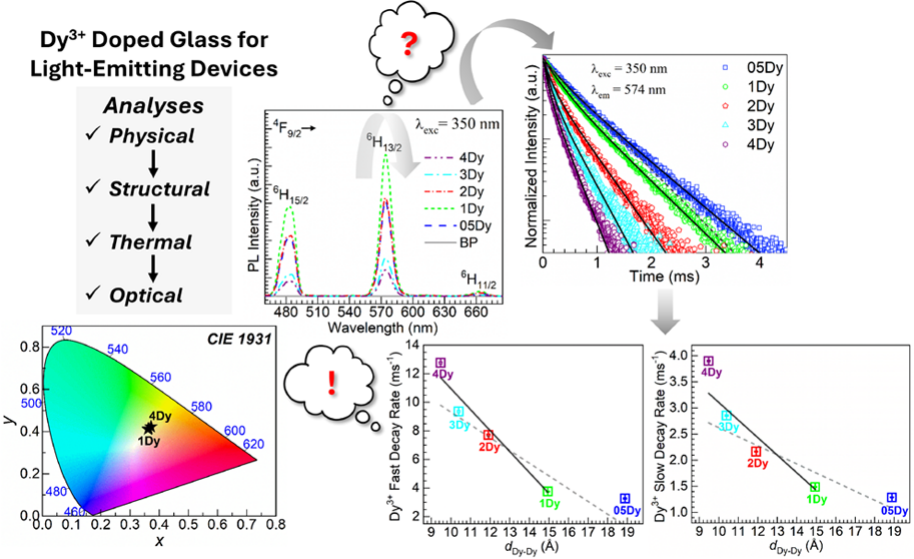

Dysprosium-doped glasses are of interest for applications in light-emitting devices, yet the full range of effects of Dy^3+^ ions on glass properties is not fully understood. In this work, phosphate glasses with 50P_2_O_5_-(50 – *x*)BaO-*x*Dy_2_O_3_ (0 ≤ *x* ≤ 4.0 mol %) nominal compositions were prepared by melting and the impact of Dy^3+^ ions on glass physical, structural, thermo-mechanical, and optical properties was evaluated. Following refractive index, density, and X-ray diffraction characterizations, the glasses were studied comprehensively through Raman spectroscopy, X-ray photoelectron spectroscopy, dilatometry, optical absorption, and photoluminescence (PL) spectroscopy. The thorough investigation and data analyses shed light on the Dy^3+^-driven structural and thermal properties reported here for the first time. The thermal expansion behavior was put in context with the reported data for other lanthanides and analyzed in the framework of the high ionic field strengths, leading to tighter glass networks. Further, a detailed analysis of the absorption, PL, and emission decay curves was carried out, providing insights into the origin of the optical behavior. Supported is the hypothesis that the cross-relaxation channels between Dy^3+^ ions taking place at low concentrations are responsible for the decrease in the decay times while the PL attractive for lighting applications still improves. Conversely, high Dy^3+^ concentrations facilitate the emission quenching proceeding via an electric dipole–dipole interaction likely incorporating the resonant excitation migration pathway for Dy^3+^–Dy^3+^ mean distances shorter than ∼15 Å.

## 1 Introduction

Research on glasses containing Dy^3+^ ions has been advancing given the interest in optical applications such as lasers and white light-emitting devices.^1−14^ Regarding glass matrices, phosphate-based hosts are known for their high metal solubility, suitable optical- and thermo-mechanical properties, low-melting character, and manufacturability, which makes them highly desirable for embedding lanthanides.^1,2,4,7,8,10−12,14,15^ Many reports on such types of glasses in the literature^1,2,4,7,12,14^ naturally focus on the experimentally determined optical qualities of the Dy^3+^-doped glasses often coupled with calculations (e.g., oscillator strengths, radiative transition probabilities) relevant for optical applications. Nonetheless, comprehensive analyses encompassing a holistic material perspective are necessary from a practical standpoint to facilitate the integration of glasses into functional devices. The work of Kaur et al.^8^ reported broadly on the thermal, structural, and optical properties of Dy^3+^-doped soda-lime aluminophosphate glasses, which were studied by various techniques including X-ray diffraction (XRD), differential scanning calorimetry, Fourier transform-infrared (FT-IR) spectroscopy, and Raman scattering in addition to optical spectroscopy. Nevertheless, thermo-mechanical properties informing about thermal expansion characteristics were not reported.^8^ Shoaib et al.^10^ added a structural characterization component via Raman spectroscopy to their optical study of Dy^3+^-doped lithium–gadolinium phosphate glasses, but the work still did not incorporate thermal analyses. In their spectroscopic study of Dy^3+^-doped potassium–zinc–calcium phosphate glasses, Mahamuda et al.^11^ reported XRD and FT-IR spectra for an undoped host only, thus precluding an evaluation of the impact of Dy^3+^ ions on the glass structure. Maheshwari and Rao^12^ studied photoluminescence (PL) downshifting of Dy^3+^-doped barium–zinc–lithium phosphate glasses and likewise reported XRD and FT-IR data for the undoped matrix. However, an evaluation of thermal properties was not conducted in refs (11) and (12). In their recent work, Jlassi et al.^14^ reported on the optical properties of Dy^3+^-doped zinc–lead–sodium phosphate glasses and provided Raman spectroscopy data limited to a specific Dy^3+^ concentration, while thermal properties were not assessed. Considering such examples from the literature, it is noticed that comprehensive studies involving a multitude of techniques are still desirable for investigating a variety of physicochemical properties linking the effects of varying Dy^3+^ ion concentrations in phosphate glasses.

Recently, our group reported a series of comprehensive structure–property studies evaluating physical, structural, thermal, and optical properties of phosphate glasses with 50P_2_O_5_-(50 – *x*)BaO-*x*Ln_2_O_3_ compositions where Ln = Eu,^16^ Nd,^17^ and Gd.^18^ The binary barium phosphate matrix has been considered as the starting point given the suitability of the large-radius Ba^2+^ cations for helping attain desirable optical, thermal, and mechanical properties adequate for various photonic applications.^2,12,15^ An additional evaluation encompassing the 50P_2_O_5_-46BaO-4Ln_2_O_3_ composition with Ln = Nd, Gd, and Yb was also performed focusing on thermal expansion effects and the structural origins of the trends guided by the high ionic field strengths of the lanthanide ions.^19^ In the present work, the investigation is extended to Dy(III) by performing a holistic physicochemical analysis seeking insights into the Dy_2_O_3_ concentration dependence on a variety of properties ultimately converging on the light-emitting qualities. The glasses were made with 50P_2_O_5_-(50 – *x*)BaO-*x*Dy_2_O_3_ (*x* = 0, 0.5, 1.0, 2.0, 3.0, and 4.0 mol %) nominal compositions by the melt-quenching technique. Thereafter, a wide-ranging experimental investigation was pursued involving the following: (i) refractive index and density measurements for basic physical properties appraisal; (ii) XRD, X-ray photoelectron spectroscopy (XPS), and Raman spectroscopy as means for structural characterization; (iii) dilatometry for the analysis of thermo-mechanical properties; (iv) optical absorption encompassing Judd–Ofelt analysis; and (v) PL spectroscopy with colorimetric analysis, radiative transition probability calculations, and emission dynamics assessment. The various parameters extracted from measurements were consequently examined in the context of the glass structure and differing Dy^3+^ concentrations, aiming to provide a broad yet detailed view of the physicochemical behavior.

## 2 Experimental Section

### 2.1 Glass Synthesis

**Figure 1:**
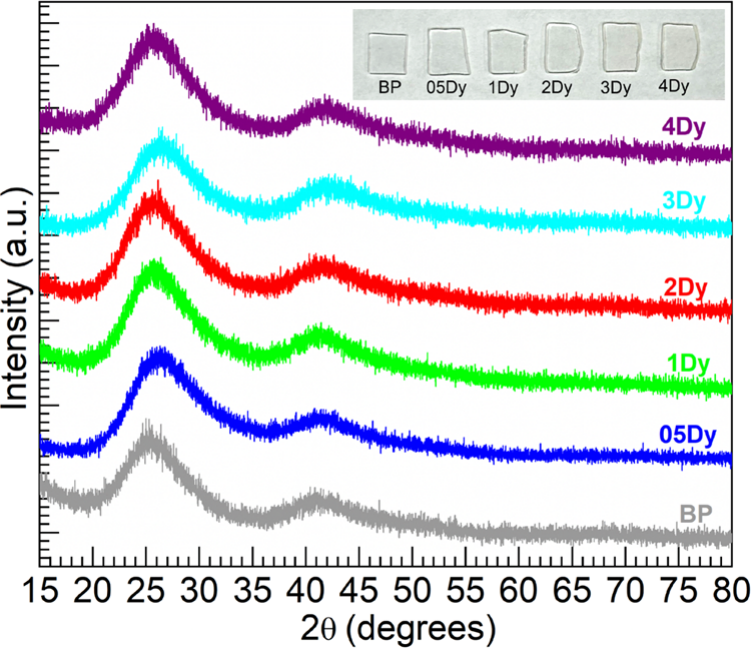
X-ray diffractograms obtained for the different glasses; the inset is a photograph of samples of the different glasses.

The glasses were prepared by melting with 50P_2_O_5_-(50 – *x*)BaO-*x*Dy_2_O_3_ nominal compositions, where *x* = 0, 0.5, 1.0, 2.0, 3.0, and 4.0 mol %. The raw materials used were P_2_O_5_ (Thermo Scientific, 98%), BaCO_3_ (Thermo Scientific, 99.8%), and Dy_2_O_3_ (Thermo Scientific, 99.9%). The compounds were weighed in appropriate quantities (about 25 g batches), thoroughly mixed, and melted under an ambient atmosphere in porcelain crucibles at 1150 °C for 20 min. The melts were swirled at ∼7 and ∼15 min to ensure homogeneity prior to being quenched onto heated steel molds. The glasses were annealed at 420 °C for 3 h to relinquish stress. The glasses were cut and polished to about 1 mm thick slabs for spectroscopic measurements. All glasses had a transparent and colorless appearance (for a photograph of some samples, see the inset of Figure 1). Glass samples were also quenched in cylindrical shapes and cut to a length (*L*) of about 2.54 cm for the dilatometric measurements. The glass labels and individual nominal molar compositions are summarized in Table 1.

**Table 1:** Glass Codes and Nominal Compositions of the 50P_2_O_5_-(50 – *x*)BaO-*x*Dy_2_O_3_ (*x* = 0, 0.5, 1.0, 2.0, 3.0, 4.0 mol %) Glasses Synthesized

glass	P_2_O_5_ (mol %)	BaO (mol %)	Dy_2_O_3_ (mol %)
BP	50.0	50.0	
05Dy	50.0	49.5	0.5
1Dy	50.0	49.0	1.0
2Dy	50.0	48.0	2.0
3Dy	50.0	47.0	3.0
4Dy	50.0	46.0	4.0

### 2.2 Measurements

#### 2.2.1 Refractive Index and Density

Refractive index (*n*_D_) measurements were performed at room temperature (RT) with an Abbe refractometer (ATAGO’s NAR-1T SOLID) using a light-emitting diode approximating the wavelength of the D-line with bromonaphthalene as the contact liquid.

The glass densities were measured by the Archimedes principle with a Mettler-Toledo XSR Analytical Balance using distilled water as the immersion liquid. The determinations were done at RT in triplicate, and the averages were reported. Additional parameters judged useful for characterizing the glasses were also calculated in accordance with corresponding formulas reported elsewhere.^16−18^

#### 2.2.2 XRD

Powder XRD was carried out at RT for amorphous nature confirmation (pulverizing glasses by mortar and pestle) with a PANalytical Empyrean X-ray diffractometer using Cu-*K*_α_ radiation (λ = 1.5406 Å). The acceleration voltage and current used were 45 kV and 40 mA, respectively.

#### 2.2.3 Raman Spectroscopy

Raman spectra were collected at RT using polished glass slabs with a Thermo Scientific DXR Raman microscope equipped with a 532 nm laser operating at a power of 10 mW. A 10× MPlan objective was employed for data collection, with the acquisition time for each spectrum set at 100 s. The estimated spectral resolution was 2.7–4.1 cm^–1^. Baseline subtraction was done using OriginPro, after which the spectra were normalized for comparison.

#### 2.2.4 XPS

XPS measurements were performed at RT with a Thermo K-Alpha XPS system using a monochromatic Al-*K*_α_ X-ray source (1486 eV). A Flood gun (low energy ionized argon beam) was used to neutralize charging effects. The adventitious carbon (C 1s) peak at 284.8 eV was used for peak position determinations. Samples were cleaned with an Ar^+^ sputter to remove surface contamination. Fitting procedures using Gaussian–Lorentzian peak shapes were employed for evaluating individual O 1s contributions.

#### 2.2.5 Thermal Analysis

Dilatometry measurements were carried out on glasses as rods in an Orton dilatometer (Model 1410B) operating at a heating rate of 3 °C/min. The data generated was used for the determination of the coefficient of thermal expansion (CTE), the glass transition temperature (*T*_g_), and the dilatometric or softening temperature (*T*_s_).

#### 2.2.6 Optical Absorption and PL Spectroscopy

Optical absorption measurements were performed at RT on ∼1 mm thick glass samples with a UV–vis–NIR Agilent Cary 5000 double-beam spectrophotometer. The reference during the measurements was air.

PL emission and excitation spectra were collected at RT under static conditions with a Horiba Fluorolog-QM spectrofluorometer by using a continuous illumination Xe lamp and a step size of 1 nm. In addition, a Xe flash lamp (pulse width of ∼2 μs) was employed for recording emission decay curves with excitation and emission conditions later specified.

## 3 Results and Discussion

### 3.1 Refractive Index, Density, and Basic Physical Properties

The refractive indexes and densities measured for the different glasses are presented in Table 2 along with other physical parameters calculated using the corresponding equations.^16−18^ The refractive indexes are observed to increase continuously with Dy_2_O_3_ replacing BaO up to a value of 1.6120 for the 4Dy glass. This type of tendency for the refractive index to rise with dysprosium concentration follows an increased polarizability and is consistent with other reports on different Dy-doped phosphate glasses.^7,10,11,14,20^ Concerning the densities, it is seen in Table 2 that the 05Dy glass density of 3.693 g/cm^3^ is marginally below the density of the BP host, which hints at a slightly higher molar volume. However, the densities increased for the 05–4Dy glasses, leading to the highest density obtained for the 4Dy glass at 3.823 g/cm^3^. The effect of increasing density goes along with the higher atomic mass of dysprosium and is analogous to the reported for the glass system wherein barium was replaced with other lanthanides.^16−18^ Further, a general trend of the density rising with Dy_2_O_3_ content is consistent with other reports for different Dy-doped phosphate glasses.^7,10−12,14,20^ The average molar masses in Table 2 increase steadily as anticipated, namely, within the 147.64–156.42 g/mol range. The molar volumes in general exhibit an increasing trend, with the highest being for the 4Dy glass with 40.91 cm^3^/mol. However, these show some slight fluctuations given their dependence on both the molar masses and the densities.^16−18^ With increasing Dy_2_O_3_ contents in the 05–4Dy glasses, the Dy^3+^ concentrations then vary in the range of 1.495 × 10^20^ to 11.78 × 10^20^ ions/cm^3^. The mean Dy^3+^–Dy^3+^ interionic distances then decrease significantly, namely, from 18.88 Å in the 05Dy glass to 9.47 Å in the 4Dy glass. These will become a point of focus when the Dy^3+^ emission output and excited-state lifetimes are evaluated in the context of ion–ion interactions.

**Table 2:** Refractive Indexes, Densities, and Parameters Related to the Basic Physical Properties of the Different Glasses

parameter	BP	05Dy	1Dy	2Dy	3Dy	4Dy
refractive index (*n*_D_)	1.5930	1.5943	1.5995	1.6067	1.6112	1.6120
density, ρ (g/cm^3^)	3.700	3.693	3.727	3.736	3.804	3.823
average molar mass, *M*_av_ (g/mol)	147.64	148.73	149.83	152.03	154.23	156.42
molar volume, *V*_m_ (cm^3^/mol)	39.90	40.28	40.20	40.69	40.54	40.91
Dy^3+^ concentration, *N*_Dy_ (×10^20^ ions/cm^3^)		1.495	2.996	5.919	8.913	11.78
Dy^3+^–Dy^3+^ mean distance, *d*_Dy–Dy_ (Å)		18.88	14.94	11.91	10.39	9.47

### 3.2 Structural Analysis

#### 3.2.1 XRD

Our previous studies performed on barium phosphate glasses with different lanthanides have shown that the glasses are X-ray amorphous within the concentration ranges studied.^16−19^ Herein, the X-ray diffractograms obtained within the 15° ≤ 2θ ≤ 80° range using Cu-*K*_α_ radiation for the glasses studied are shown in Figure 1 (the photograph in the inset shows samples of the different glasses as slabs). The different samples consistently display broad qualitative features characterizing long-range structural disorder. Additionally, the XRD patterns have no crystallization peaks which supports the noncrystalline nature of the glasses prepared.

#### 3.2.2 Raman Spectroscopy

**Figure 2:**
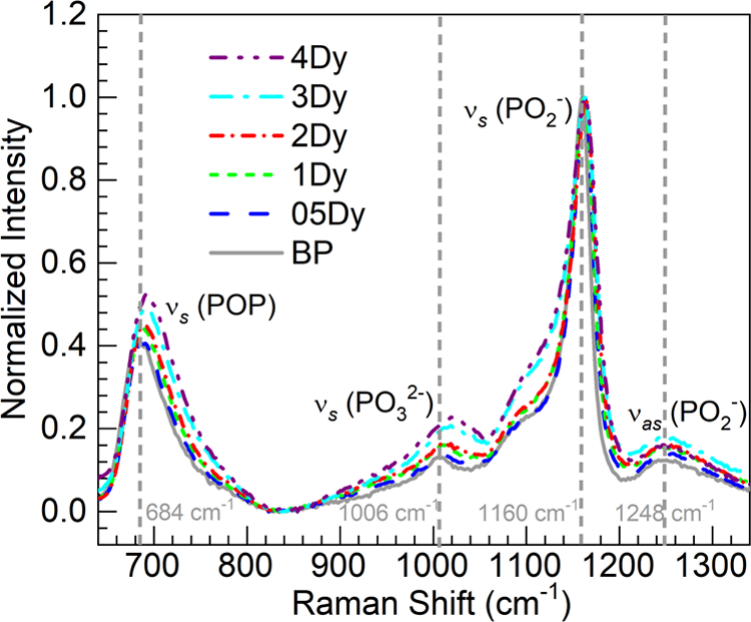
Normalized Raman spectra for the various glasses within the 640–1340 cm^–1^ spectral range for comparison; the main spectroscopic features in the BP glass as reference are indicated (vertical dashed lines—wavenumbers displayed).

Figure 2 shows the Raman spectra for the 05–4Dy glasses together with the BP host as the reference, which were all normalized with respect to the strongest band within the 640–1340 cm^–1^ range. The different features observed are typical of phosphate glasses and were thus assigned in accord with previous works.^16−19,21,22^ The BP glass host taken as reference presents toward the low energy region a band around 684 cm^–1^ recognized as the in-chain symmetric stretching vibrations in P–O–P bridges, ν_s_(POP), in *Q*^2^ units (PO_4_ tetrahedra with 2 bridging oxygens, BOs). The small feature observed at about 1006 cm^–1^ is credited to the symmetric stretch, ν_s_(PO_3_^2–^), in nonbridging oxygens (NBOs) concerning *Q*^1^ units (PO_4_ tetrahedra with 1 BO). Then the binary BP host shows the most intense band around 1160 cm^–1^ due to the out-of-chain symmetric stretch in the PO_2_^–^ groups, ν_s_(PO_2_^–^), occurring within the NBOs of the *Q*^2^ units. Lastly, the corresponding asymmetric stretching vibrations, ν_as_(PO_2_^–^), are expressed at around 1248 cm^–1^. The most energetic vibrations, the stretching vibrations, are mainly responsible for radiationless processes of luminescent ions when embedded. Hence, the Raman assessment is in good agreement with the average phonon energy generally accepted for phosphate glasses around 1200 cm^–1^.^15^

The Raman spectra for the 05–2Dy glasses in Figure 2 appear similar to the BP host, suggesting that comparable glass networks were attained for low Dy_2_O_3_ content. On the other hand, the spectra for the 3Dy and 4Dy glasses manifest increased intensity with broadening toward the lower frequency wing of the ν_s_(PO_2_^–^) band as well as the ν_s_(PO_3_^2–^) band. This suggests that a depolymerization effect was induced at high Dy_2_O_3_ contents which is especially noticeable by the increased ν_s_(PO_3_^2–^) feature characteristic of *Q*^1^ units. Additionally, toward the low-frequency region, the ν_s_(POP) band shows some differences more distinctly for the 3Dy and 4Dy glasses, for instance concerning position, width, and intensity relative to the ν_s_(PO_2_^–^) band. These aspects appear consistent with a somewhat larger number of shorter chains being realized as the concentration of Dy^3+^ increased considerably. Nonetheless, the effects appear less pronounced than that observed for the glass system with Eu^3+^,^16^ Nd^3+^,^17^ and Gd^3+^.^18^ To evaluate these effects more closely, presented in Table 3 are the peak positions and the full-width at half-maximum (FWHM) values of the ν_s_(POP) band (BO-related) as well as the ν_s_(PO_2_^–^) band (NBO-related), alongside the intensity ratios (*I*_NBO_/*I*_BO_) as a parameter of interest.^18,21^ Both bands exhibit increased broadening with the Dy_2_O_3_ content, especially noticeable for the 3Dy and 4Dy glasses. The ν_s_(PO_2_^–^) band stemming from NBOs however exhibits minor variations in band position, whereas the ν_s_(POP) band shifts continuously toward a higher wavenumber as it broadens. This latter effect is indicative of the presence of shorter PO_4_ tetrahedra chains which yield higher frequency components and bring about band asymmetry toward higher frequencies.^18,19,21,22^ The *I*_NBO_/*I*_BO_ ratios are then seen in Table 3 to generally decrease except for the 05Dy glass, which appeared marginally above the BP host. The lowering trend of the *I*_NBO_/*I*_BO_ ratios has been also linked to depolymerization effects induced by the cationic modifiers.^16,18,21^

**Table 3:** Spectral Positions and Full-width at Half-Maximum (FWHM) Values for the Bridging Oxygen (BO)-ν_s_(POP) and Nonbridging Oxygen (NBO)-ν_s_(PO_2_^–^) Raman Bands, and the NBO to BO Band Intensity Ratio, *I*_NBO_/*I*_BO_, Obtained for the Different Glasses

	BO-ν_s_(POP) band		NBO-ν_s_(PO_2_^–^) band		
glass	peak (cm^–1^)	FWHM (cm^–1^)	peak (cm^–1^)	FWHM (cm^–1^)	*I*_NBO_/*I*_BO_
BP	684	56	1160	28	2.42
05Dy	686	58	1162	30	2.47
1Dy	689	58	1162	33	2.27
2Dy	690	60	1163	35	2.22
3Dy	691	64	1162	43	2.03
4Dy	693	65	1162	45	1.90

**Figure 3:**
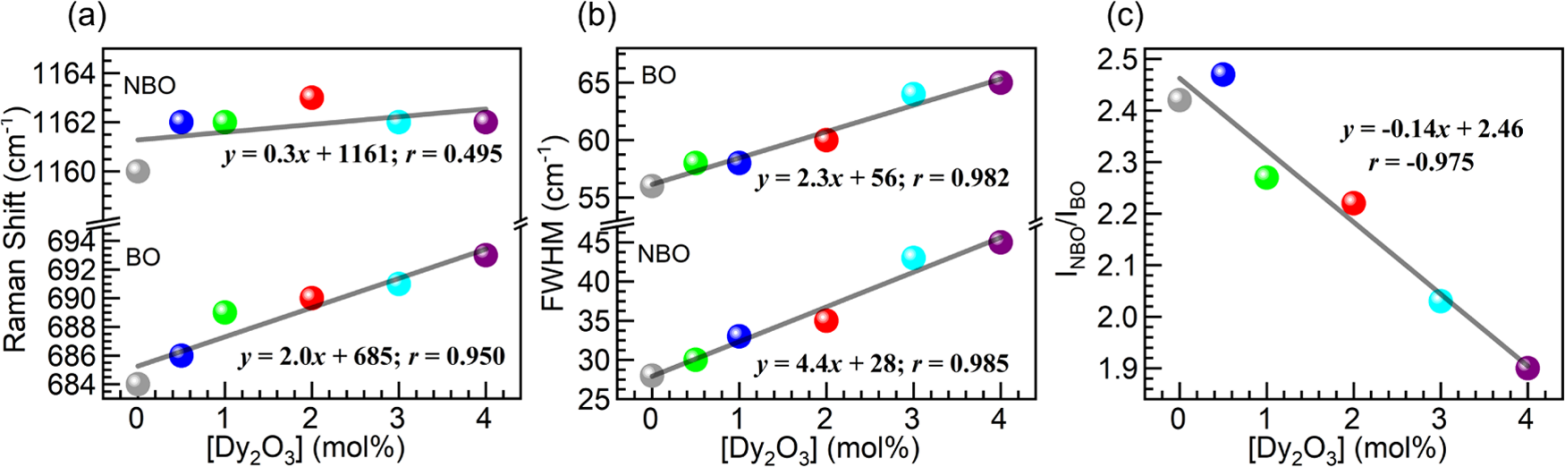
Plots of Raman spectral parameters for the bridging oxygen (BO) band, ν_s_(POP), and the nonbridging oxygen (NBO) band, ν_s_(PO_2_^–^), as a function of the Dy_2_O_3_ concentration (mol %) in the glasses: (a) band position; (b) full-width at half-maximum (FWHM); and (c) the intensity ratio, *I*_NBO_/*I*_BO_. The solid lines are linear fits to the data (sphere symbols); equations and correlation coefficients (*r*) are displayed.

To graphically assess the influence of dysprosium content on the Raman spectra, plots in Figure 3a–c show the position and FWHM values of both the ν_s_(POP) and ν_s_(PO_2_^–^) bands, along with the *I*_NBO_/*I*_BO_ ratios as a function of the Dy_2_O_3_ concentration. The data sets were subjected to linear regression analyses (solid lines in all panels of Figure 3), yielding the equations and correlation coefficients displayed therein. The position of the ν_s_(POP) band in Figure 3a clearly shows a trend toward higher frequencies with increasing Dy_2_O_3_ concentrations. The intercept deduced from the fit of 685 cm^–1^ is in good agreement with the value for the BP glass of 684 cm^–1^ (Table 3). Conversely, the ν_s_(PO_2_^–^) band position exhibits fluctuations and lacks correlation, although the intercept of 1161 cm^–1^ obtained from the fit is close to that for the host (Table 3). On the other hand, the FHWM values of both the BO-related ν_s_(POP) and NBO-related ν_s_(PO_2_^–^) bands are observed in Figure 3b to increase with Dy_2_O_3_ concentration. The intercepts in this case also coincide with the BP host values shown in Table 3. The fit to the data of the *I*_NBO_/*I*_BO_ ratio yielded an intercept of 2.46 °C closer to the 05Dy glass than to the BP host (Table 3). The observed trends fairly resemble those reported for the glasses containing Eu^3+^^16^ and Gd^3+^^18^ in the barium phosphate glass system. In such instances, the changes were interpreted in terms of depolymerization effects induced by the lanthanide ions.^16,18^ The depolymerization of the glass matrix was further supported by O 1s XPS analysis for Gd^3+^ and Nd^3+^ in the barium phosphate glasses containing 4 mol % of the oxides.^19^ We proceeded then in the present investigation with an oxygen speciation analysis by XPS in the 05–4Dy glasses under consideration to corroborate the structural modifications.

#### 3.2.3 XPS

**Figure 4:**
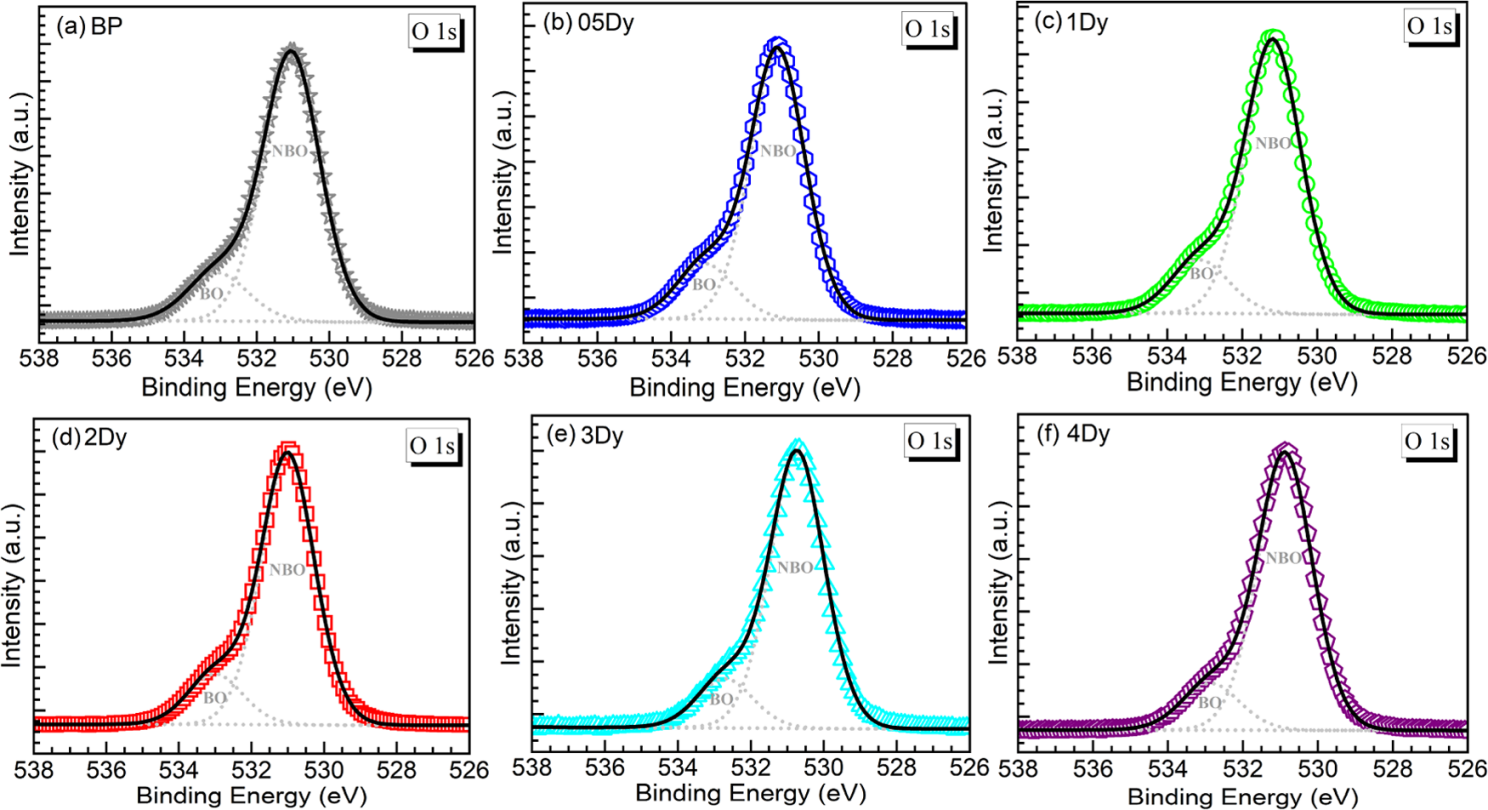
XPS O 1s peaks registered for the various glasses studied (open symbols): (a) BP; (b) 05Dy; (c) 1Dy; (d) 2Dy; (e) 3Dy; and (f) 4Dy. The spectra are deconvoluted into the bridging oxygen (BO) and nonbridging oxygen (NBO) species (dotted curves; results in Table 4). The cumulative fits are the solid traces.

The spectra obtained for the different glasses from the O 1s XPS measurements are shown in Figure 4a–f. The experimental spectra all exhibit the O 1s peaks around 531 eV with a shoulder toward the high binding energy (BE) side. The dominant peak reflects the presence of terminal NBOs (P–O^–^) interacting with the metal cations, whereas the shoulder indicates the presence of BOs (P–O–P) in the glass network.^19,23−25^ The spectra were consequently deconvoluted into the two oxygen contributions as previously performed.^19^ The resulting bands are also presented overlaid with the experimental traces and the cumulative fits in each panel in Figure 4. The corresponding parameters of BE, FWHM, and % relative area are given in Table 4. As a main parameter deduced from the areas of the two bands, the relative amounts of BO and NBOs were estimated for the glasses. As seen in Table 4, the NBO percentage first decreased for the 05Dy glass relative to the host but then increased for the 1Dy and 2Dy glasses. The latter exhibited a NBO content of 83.8% comparable to the undoped BP glass with 83.6%. These results seem consistent with the Raman spectra (*vide supra*) wherein less significant variation was seen among the BP and 05–2Dy glasses. On the other hand, the 3Dy and 4Dy glasses yielded higher NBO contents of 84.7 and 84.9%, respectively. The analysis of the O 1s spectra then supports that shorter chain lengths were produced for the highest Dy_2_O_3_ concentrations. This means that some degree of depolymerization occurred for the 3Dy and 4Dy glasses, which harmonizes with the Raman assessment evidencing more significant variation in the spectral parameters for such glasses. Nevertheless, the depolymerization effect was not as dramatic as indicated for the barium phosphate glasses containing 4 mol % Nd_2_O_3_ (NBO = 87.3%) or 4 mol % Gd_2_O_3_ (NBO = 86.6%).^19^ Still, the impact perceived for the 4Dy glass with 4 mol % Dy_2_O_3_ increasing the NBO content (NBO = 84.9%) is noticeable in contrast to the case with 4 mol % Yb_2_O_3_ substituting for BaO (NBO = 77.8%)^19^ in the glass wherein depolymerization was not supported.

**Table 4:** O 1s Binding Energy (BE), Full-Width at Half-Maximum (FWHM), and % Relative Area for the Different Oxygen (BO—Bridging Oxygen; NBO—Nonbridging Oxygen) Components in the Various Glasses as Estimated from Decomposing the XPS Spectra

		O 1s XPS		
glass	component	BE (eV)	FWHM (eV)	% area
BP	BO	533.1	2.0	16.4
	NBO	531.0	1.8	83.6
05Dy	BO	533.1	1.9	17.9
	NBO	531.1	1.8	82.1
1Dy	BO	533.1	1.9	17.2
	NBO	531.2	1.8	82.8
2Dy	BO	533.0	1.9	16.2
	NBO	531.0	1.8	83.8
3Dy	BO	532.7	1.8	15.3
	NBO	530.7	1.8	84.7
4Dy	BO	532.9	1.8	15.1
	NBO	530.9	1.7	84.9

The outcome of Gd(III) and Nd(III) causing significant disruption of the glass network was rationalized in terms of the ionic size effects driving the ionic field strengths underpinning the thermal behavior.^17−19^ We may then consider that assuming 6-fold coordination^26^ the ionic radius of Dy^3+^, *R*_Dy(III)_, according to Shannon^27^ is 0.912 Å, whereas for Nd^3+^, Gd^3+^, and Yb^3+^ the values are *R*_Nd(III)_ = 0.983 Å, *R*_Gd(III)_ = 0.938 Å and *R*_Yb(III)_ = 0.868 Å, correspondingly. It thus seems that the degree of network disruption follows the ionic size trends *R*_Nd(III)_ > *R*_Gd(III)_ > *R*_Dy(III)_ > *R*_Yb(III)_. Henceforth, we consider results from the thermal/dilatometric characterization performed on the Dy^3+^-containing glasses expanding on the structural assessment.

### 3.3 Thermal/Dilatometric Properties

**Figure 5:**
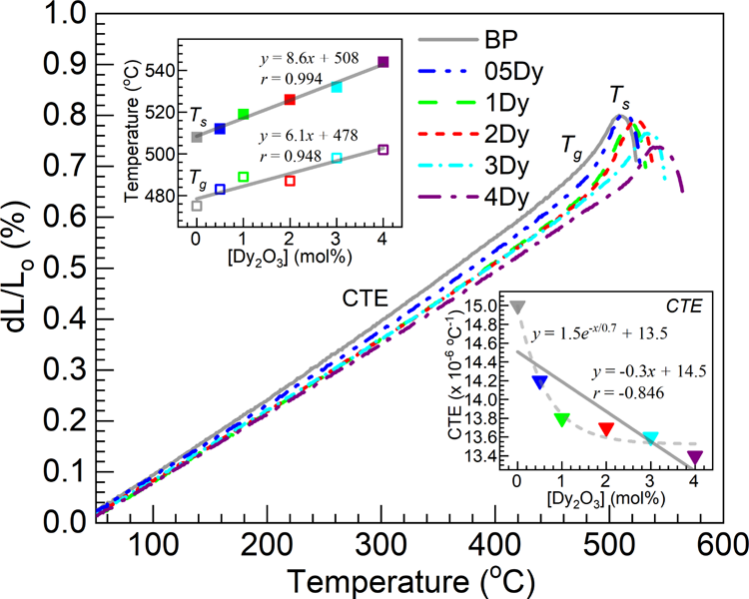
Dilatometric profiles obtained for the different glasses; estimated values of softening temperature (*T*_s_), glass transition temperature (*T*_g_), and coefficient of thermal expansion (CTE) are summarized in Table 5. The top inset is a plot of *T*_s_ (filled squares) and *T*_g_ (open squares) vs Dy_2_O_3_ concentration in the glasses; the bottom inset plots the CTE values (filled inverted triangles) vs. the Dy_2_O_3_ concentration. The solid lines are linear fits to the data (equations and correlation coefficients, *r*, displayed) and the dashed trace in the bottom inset is an exponential fit (equation displayed).

Figure 5 shows the dilatometric profiles obtained for the 05–4Dy glasses together with the undoped glass as a reference. The thermal expansion profiles were used to estimate the complete set of parameters to include the *T*_s_, *T*_g_, and CTE (within 50–400 °C) following the traditional approach.^19,25^ The results are summarized in Table 5. The different values are plotted for a graphical appraisal in the top and bottom insets of Figure 5 as a function of the Dy_2_O_3_ concentration (mol %) in the glasses. It is observed that the *T*_s_ and *T*_g_ values tend to increase with increasing Dy_2_O_3_ contents, whereas the CTE values decrease continuously. Similar trends with regards to thermal behavior assessed by dilatometry (and calorimetry for *T*_g_) have been observed for barium phosphate glasses containing increasing amounts of Eu_2_O_3_,^16^ Nd_2_O_3_,^17^ and Gd_2_O_3_.^18^

**Table 5:** Thermal Parameters of Softening Temperature (*T*_s_), Glass Transition Temperature (*T*_g_), and Coefficient of Thermal Expansion (CTE) Are Estimated for the Different Glasses from Dilatometry (Figure 5)

glass	*T*_s_ ± 3 (°C)	*T*_g_ ± 4 (°C)	CTE ± 0.1 (×10^–6^ °C^1–^)
BP	508	475	15.0
05Dy	512	483	14.2
1Dy	519	489	13.8
2Dy	526	487	13.7
3Dy	532	498	13.6
4Dy	544	502	13.4

The data concerning the steady rise in *T*_s_ with increasing Dy_2_O_3_ concentration was subjected to regression analysis as shown in the top inset of Figure 5 (filled squares). It yielded a correlation coefficient *r* of 0.994 indicating a strong linear correlation, with an intercept of 508 °C coinciding with the *T*_s_ of the undoped host (Table 5). The *T*_g_ values in Table 5 exhibit fluctuation midrange in going from the 1Dy to the 2Dy glass, which is reasonable given the relatively high error associated (about ±4 °C^25^). Still, an overall trend of an increase in the *T*_g_ is supported. Thus, linear regression analysis was performed, as shown in the top inset of Figure 5 (unfilled squares). Here the correlation coefficient of 0.948 obtained was not as strong as that with the *T*_s_. Nevertheless, the intercept of 478 °C is in good agreement with the *T*_g_ of the BP glass of 475 °C (Table 5). The obtained slope of 6.1 °C/mol % was also somewhat lower than the slope of 8.6 °C/mol % stemming from the regression analysis on the *T*_s_ data. The softening point thus appears to be more sensitive to the change in Dy_2_O_3_ concentration. Concerning the decreasing CTE values, the linear regression analysis of the data in the bottom inset of Figure 5 did not exhibit a linear correlation (*r* = −0.846). Instead, an exponential decay function appeared to provide a better fit.

Reports on the influence of Dy^3+^ ions on the thermo-mechanical properties of phosphate glasses are currently lacking, while those concerned with other glass matrices are still limited. In their dilatometric evaluation of Dy^3+^-doped germano-borate multicomponent glasses with Dy_2_O_3_ added within 40–75 wt %, Mollaee et al.^28^ observed increasing *T*_g_ values but these were accompanied by an upward trend in the CTE from 61.8 to 94.1 × 10^–7^ °C^1–^. Linganna et al.^29^ studied GeO_2_–B_2_O_3_–Al_2_O_3_-(15 – *x*)Ga_2_O_3_–SiO_2_-*x*Dy_2_O_3_ compositions and reported increasing dilatometric temperatures with Dy_2_O_3_ content with fluctuations in the CTE values in the 4.72–5.06 × 10^–6^ °C^1–^ range. The authors pointed out that there was a propensity for Dy^3+^ to lower the CTE, which was advantageous for the application in magneto-optical devices.^29^ Sanyal et al.^30^ studied Dy^3+^-doped lithium borate glasses for thermoluminescence dosimetry and noticed that the CTE of the undoped glass was higher, thus suggesting an increase in rigidity with addition of Dy_2_O_3_. In this study, the rising trends in *T*_s_ and *T*_g_ observed (Table 5) are consistent with a glass strengthening effect realized with Dy^3+^ ions. The analogous trends that were observed with other lanthanides in the barium phosphate glass system were interpreted as being driven by the high ionic field strengths of the lanthanide ions.^16−18^ Hence, we may herein consider that the ionic radii for Ba^2+^ with expected coordination number of eight^31^ is *R*_Ba(II)_ = 1.42 Å,^27^ whereas *R*_Dy(III)_ = 0.912 Å^27^ for Dy^3+^ assuming 6-fold coordination.^26^ Calculating the ionic field strengths (*F*_*i*_) with the common formula^17,18^
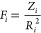
1where *Z*_*i*_ is the cation charge that gives values of *F*_Ba(II)_*=* 0.992 Å^–2^ and *F*_Dy(III)_*=* 3.607 Å^–2^ for Ba^2+^ and Dy^3^, respectively. Accordingly, replacing Ba^2+^ by Dy^3+^ ions with higher ionic field strength in the phosphate glass system seems to be strengthening the phosphate network through the cationic interactions with NBOs.

The results for the CTE reported in Table 5 also showed consistency with the decrease in the values with increasing Dy_2_O_3_ content. This outcome is in harmony with reports on the CTE of phosphate glasses decreasing with increasing concentrations of Eu^3+^,^16^ Nd^3+^,^17^ and Gd^3+^.^18^ It is interesting to note that like in the prior cases,^16−18^ the CTE herein decreases with Dy^3+^ concentration even though some depolymerization was induced (*vide supra*). It is important to note that, by itself, the disruption of the glass network would tend to increase the CTE in connection with loose network connectivity.^25,32^ Hence, the manifestation of tighter networks being achieved by the lanthanide ions has been rationalized in terms of ionic field strength effects in agreement with works on other glass systems performed by different groups.^33−36^ It is therefore realistic to construe the CTE decreasing in the Dy^3+^-doped glasses is reflecting a more compact glass structure less susceptible to expansion due to the increase in the concentration of Dy^3+^ ions with high-field strength (*F*_Dy(III)_*=* 3.607 Å^–2^) compared to Ba^2+^ ions (*F*_Ba(II)_ = 0.992 Å^–2^).

**Figure 6:**
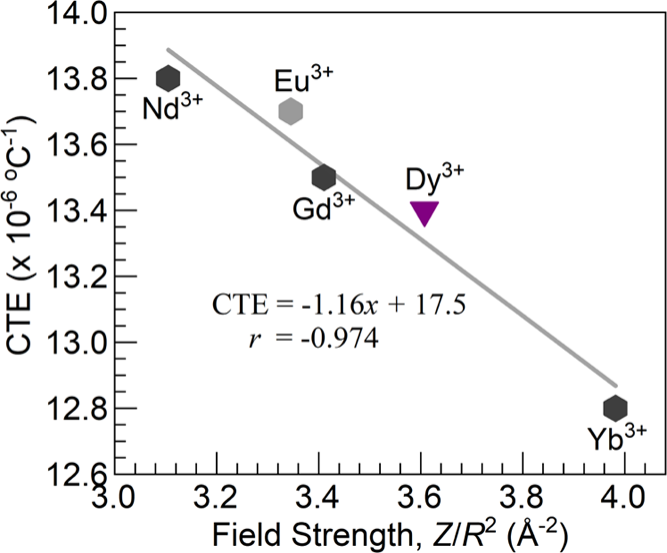
Plot of CTE as a function of the ionic field strength of the Ln^3+^ ions of various glasses of 50P_2_O_5_-46BaO-4Ln_2_O_3_ composition with Ln = Nd,^19^ Eu,^16^ Gd,^19^ Dy (this work), and Yb^19^ compositions. The solid line is a linear fit to the data (equation and correlation coefficient, *r*, displayed).

It is worth at this juncture to draw a comparison between the CTE of the 4Dy glass with 4 mol % Dy_2_O_3_ and the reported for the glasses with 4 mol % Ln_2_O_3_ with Ln = Nd, Gd, Yb^19^ and also 4 mol % Eu_2_O_3_^16^ in the phosphate glass system. It is noticed that the CTE of the 4Dy glass of 13.4 × 10^–6^ °C^1–^ in Table 5 falls in between the reported for the Gd^3+^- and Yb^3+^-containing glasses of 13.5 × 10^–6^ °C^1–^ and 12.8 × 10^–6^ °C^1–^, respectively.^19^ This agrees with the fact that the field strength for Dy^3+^, *F*_Dy(III)_*=* 3.607 Å^–2^, lies in between those for Gd^3+^ and Yb^3+^ taken for the 6-fold coordinated ions as *F*_Gd(III)_*=* 3.410 Å^–2^ and *F*_Yb(III)_*=* 3.982 Å^–2^, respectively.^19^ In Figure 6, a plot is presented of the different CTE values as a function of the ionic field strength of the lanthanide ions where we combine the current result for the 4Dy glass (inverted triangle) with the reported for various glasses (solid hexagons) of 50P_2_O_5_-46BaO-4Ln_2_O_3_ composition with Ln = Nd,^19^ Eu,^16^ Gd,^19^ and Yb^19^ compositions. The graph expands on the reported in ref (19) and shows similar values for the slope and intercept despite the lower magnitude for the correlation coefficient. It overall supports the impact of the high ionic field strength of the lanthanide ions driving the decrease in CTE similar to that reported by Menke et al.^33^ and Lofaj et al.^34^ for Si–Al–O–N and oxynitride systems, respectively (the latter lacking Dy^3+^).

### 3.4 Optical Properties

#### 3.4.1 Absorption Spectroscopy Analysis

**Figure 7:**
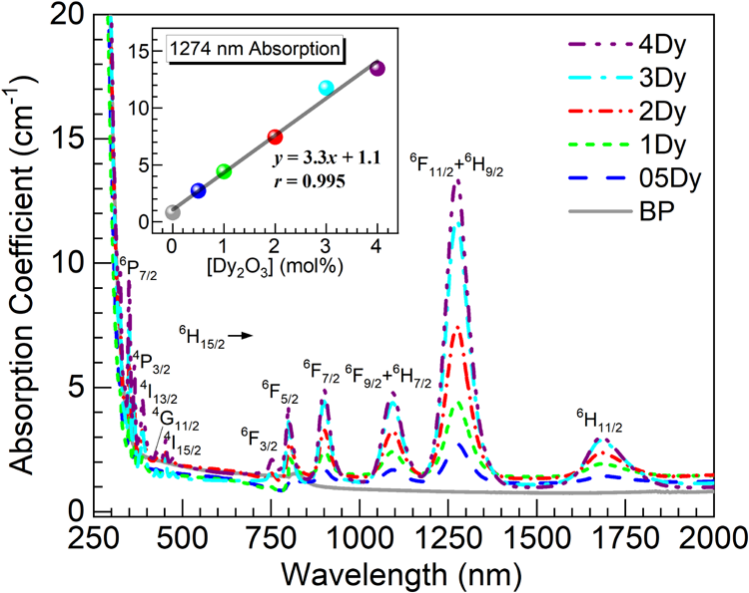
UV–vis–NIR optical absorption spectra obtained for the BP and 05–4Dy glasses. The inset is a plot of the absorption intensity at 1274 nm vs. Dy_2_O_3_ concentration in the glasses; the solid line is a linear fit to the data (equation and correlation coefficient, *r*, displayed).

Presented in Figure 7 are the UV–vis–NIR absorption spectra obtained for the glasses under consideration. Unlike the undoped glass, the absorption spectra of the 05–4Dy glasses display 11 absorption bands ascribed to ^6^H_15/2_ → ^6^P_7/2_, ^4^P_3/2_, ^4^I_13/2_, ^4^G_11/2_, ^4^I_15/2_, ^6^F_3/2_, ^6^F_5/2_, ^6^F_7/2_, ^6^F_9/2_ + ^6^H_7/2_, ^6^F_11/2_ + ^6^H_9/2_, and ^6^H_11/2_ transitions as labeled.^2,37,38^ The Dy^3+^ absorption intensity is observed to increase with Dy_2_O_3_ concentration, most prominently seen for the absorption peak at around 1274 nm in connection with the ^6^H_15/2_ → ^6^F_11/2_ + ^6^H_9/2_ transitions. In the inset of Figure 7, the peak intensity of such absorption is plotted as a function of the Dy_2_O_3_ concentration. A linear regression analysis yielded a correlation coefficient *r* of 0.995, indicating strong correlation. This supports that the increasing amounts of Dy^3+^ ions were effectively incorporated into the glass matrix as it was similarly observed for instance when embedding Nd^3+^ ions in the glass system.^17^ This may be assisted by the depolymerization effect induced by the lanthanide ions acting as network modifiers, as indicated by the Raman and O 1s XPS data.

The oscillator strength, *f*_exp_, of the absorption transitions denotes the strength of the electric and magnetic dipole transitions of the Dy^3+^ ion in the host glass matrix.^37^ Herein, the oscillator strength of 8 absorption key transitions as indicated in Table 6 were calculated by measuring the areas of the particular absorption band using the relation
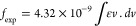
2where ε is the molar extinction coefficient and υ.*dυ* is the area under the absorption peak. The measured density, Dy^3+^ ion concentration, and glass thicknesses were used to calculate the extinction coefficients.^39^ Guided by the PL results wherein the maximum intensity was observed for the 1Dy glass (*vide infra*), the oscillator strengths of different transitions were calculated for such glass, which are summarized in Table 6. The absorption band that is considered as the hypersensitive band (^6^H_15/2_ → ^6^F_11/2_ + ^6^H_9/2_) registered the highest oscillator strength. In addition, the eight *f*_exp_ values were used to calculate the Judd–Ofelt (J–O) intensity parameters for the 1Dy glass using the least-squares fit method according to the J–O theory. The trend of J–O parameters is often used to comprehend the influence of local surroundings, local symmetry, nature of Dy–O ligand bonding, and to evaluate the radiative properties of the Dy^3+^-doped glasses.^40,41^ The necessary J–O parameters (Ω_2_, Ω_4_, Ω_6_) were determined by fitting the experimental oscillator strength (*f*_exp_) values to the following equation^37^
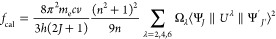
3where, *m*_e_ is the mass of electron, *c* is the speed of light in a vacuum, *n* is the refractive index of the glass, υ is the frequency (cm^–1^) of absorption associated with the absorption transition Ψ_*J*_ → Ψ′_J′_*J* and *J*^*′*^ are the total angular momentum quantum number of the ground and excited state of Dy^3+^, respectively, and *U*^λ^ is the doubly reduced matrix elements of the observed transition levels of Dy^3+^ ions, which is independent of the host material. The calculated J–O parameters of the 1Dy glass are shown in Table 7. These calculated J–O parameters were further used to compute the theoretical oscillator strength of the Ψ_*J*_ to Ψ′_*J*′_ transition levels of Dy^3+^ ions. The obtained theoretical strengths of induced electronic-dipole transition between 4f energy levels of Dy^3+^ ions are shown in Table 6. The measured oscillator strength values are consistent with the calculated values for hypersensitive absorption transitions and slightly deviated for other absorption transitions. Further, the legitimacy of the least-squares fitting procedure involved in the J–O parameter calculation was deduced by calculating the root-square-mean deviation (r.m.s.) among the oscillator strengths of the measured and computed values using the following equation^11,42^
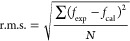
4where, *N* is the number of electronic transitions involved in the least-squares fitting in this study (equal to 8) and *f*_exp_ and *f*_cal_ are the experimental and theoretical oscillator strengths, respectively, of individual transitions. The r.m.s. deviation obtained for the 1Dy glass is 0.677 × 10^–6^ indicating good agreement between the experimental and theoretical oscillator strengths and validates the intensity of the determined J–O parameters. According to Jorgensen theory, the Ω_2_ parameter varies as a function of changes in local surroundings, such as asymmetry around the ion site and the nature of the bonding between the ion and the neighborhood ligands.^43^ In addition, Ω_4_ and Ω_6_ depend on the bulk properties of the glasses such as viscosity and rigidity. In the present case, the trend of J–O parameters as Ω_2_ > Ω_4_ > Ω_6_ for the 1Dy glass is shown in Table 7 together with reported values for other Dy^3+^-doped glasses for comparison: PKSAD10: (58.5)P_2_O_5_-17K_2_O-(14.5)SrO-9Al_2_O_3_-1.0Dy_2_O_3_;^7^ PLGD1.0: (65)P_2_O_5_-17Li_2_O-17Gd_2_O_3_-1.0Dy_2_O_3_;^10^ PDy10:55P_2_O_5_-15ZnO-19CaF_2_-1Dy_2_O_3_;^11^^11^ PDY1:60P_2_O_5_-14BaO-15ZnO-10Li_2_O-1Dy_2_O_3_;^12^^12^ NPZDy1.0:59NaPO_3_-25PbF_2_-15NaF-1.0Dy_2_O_3_;^14^ BPAPbLiDy1:44H_3_BO_3_-20P_2_O_5_-10Al_2_O_3_-10PbO-15Li_2_O_3_-1Dy_2_O_3_;^44^ TYDF1:75TeO_2_-25YF_3_-8DyF_3_;^45^^45^ BBASKDy1:30SiO_2_-11Al_2_O_3_-44B_2_O_3_-5BaO-10K_2_O-1Dy_2_O_3_;^46^^46^ and TZONbSFDy0.5:60TeO_2_-35ZnO-5SrF_2_-0.5Dy_2_O_3_.^47^^47^ It is to be observed that the JO parameters for the 1Dy glass showed significant change according to the phosphate content in the host glass network compared to other phosphate glasses.^7,10,14^ The value of Ω_2_ is lower whereas the value of Ω_6_ is higher relative to other reports^7,10,14^ likely in connection with the weaker oscillator strength of the absorption transitions in the present host glass network. This suggests a bonding environment modification around the Dy^3+^ ions in the present glass host. Namely, the lower Ω_2_ points to higher symmetry around Dy^3+^ ions^48^ whereas the higher Ω_6_ value indicates a higher degree of covalency stemming from π-electron donation from PO_4_ tetrahedra.^49^

**Table 6:** Experimental Oscillator Strength (*f*_exp_ × 10^–6^), Theoretical Oscillator Strength Included ^6^H_15/2_ → Next Levels (*f*_cal_ × 10^–6^), and Root-Mean-Square Deviation (r.m.s.) Pertaining to the 1Dy Glass

transitions	1Dy glass	
^6^H_15/2_ →	*f*_exp_ × 10^–6^	*f*_cal_ × 10^–6^
^6^P_7/2_	1.89	0.0525
^4^P_3/2_	0.76	0.4086
^4^I_13/2_	1.01	0.9927
^4^I_15/2_	0.67	0.5792
^6^F_7/2_	3.36	3.0418
^6^F_9/2_ + ^6^H_7/2_	3.85	3.9297
^6^F_11/2_ + ^6^H_9/2_	9.14	9.124
^6^H_11/2_	1.5	1.6749
r.m.s. = 0.677 × 10^–6^		

**Table 7:** Judd–Ofelt Intensity Parameters Ω_2_ (×10^–20^ cm^2^), Ω_4_ (×10^–20^ cm^2^), and Ω_4_ (×10^–20^ cm^2^) of 1Dy Glass of This Work and Comparison with Reported Values for Other Dy^3+^-Doped Glasses

glass code	Ω_2_	Ω_4_	Ω_6_	trend	reference
1Dy	7.96	3.821	3.056	Ω_2_ > Ω_4_ > Ω_6_	present work
PKSAD10	10.20	2.14	2.55	Ω_2_ > Ω_6_ > Ω_4_	(7)
PLGD1.0	10.94	3.66	2.60	Ω_2_ > Ω_4_ > Ω_6_	(10)
PDy10	6.28	1.665	2.038	Ω_2_ > Ω_6_ > Ω_4_	(11)
PDY1	11.24	1.18	0.27	Ω_2_ > Ω_6_ > Ω_4_	(12)
NPZDy1.0	10.524	9.622	0.982	Ω_2_ > Ω_4_ > Ω_6_	(14)
BPAPbLiDy1	2.91	0.90	1.0735	Ω_2_ > Ω_6_ > Ω_4_	(44)
TYDF1	10.62	1.76	3.99	Ω_2_ > Ω_6_ > Ω_4_	(45)
BBASKDy1	4.83	1.89	1.59	Ω_2_ > Ω_4_ > Ω_6_	(46)
TZONbSFDy0.5	6.47	1.38	1.66	Ω_2_ > Ω_6_ > Ω_4_	(47)

#### 3.4.2 PL Spectroscopy Assessment

**Figure 8:**
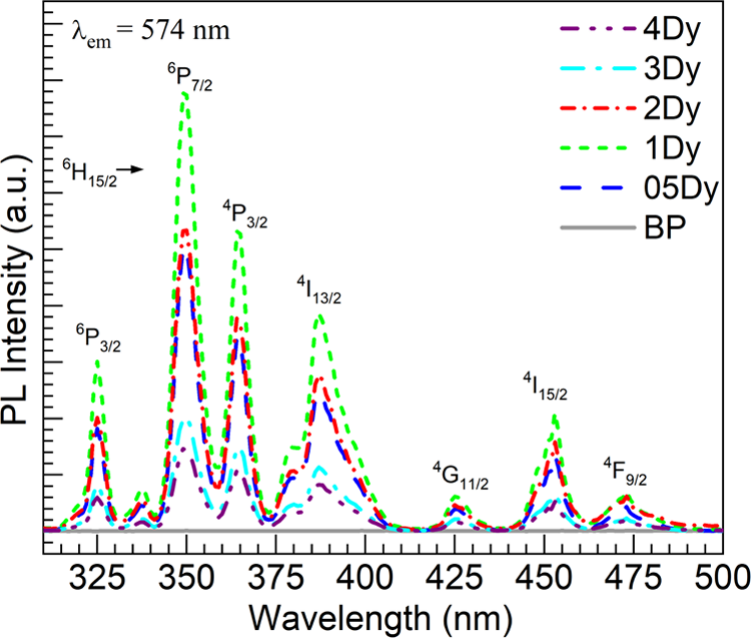
PL excitation spectra recorded for the various glasses with the detection wavelength set at 574 nm to monitor ^4^F_9/2_ → ^6^H_13/2_ emission from Dy^3+^ ions.

**Figure 9:**
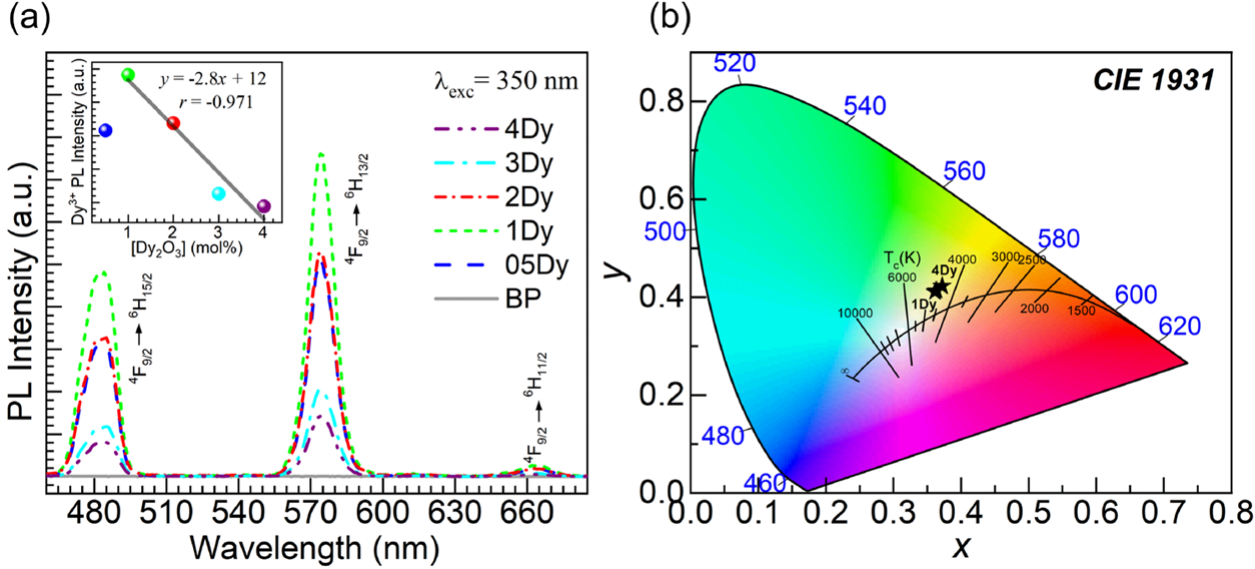
(a) Emission spectra obtained for the various glasses under excitation at 350 nm; the inset is a plot of the integrated 3-band Dy^3+^ emission intensity vs. Dy_2_O_3_ concentration in the glasses; the solid line is the linear fit to the data for the 1–4Dy glasses (equation and correlation coefficient, *r*, displayed). (b) CIE 1931 chromaticity diagram with coordinates (star symbols; complete *x*,*y* coordinates and correlated color temperatures are reported in Table 8) of emission color calculated for the Dy^3+^-doped glasses from the spectra in panel (a).

We begin this section with the PL excitation spectra shown in Figure 8 which were recorded for the glasses with detection wavelength set at 574 nm pertaining to the key ^4^F_9/2_ → ^6^H_13/2_ emission from Dy^3+^ ions.^10,20,50^ This illustrates the Dy^3+^ PL intensity evolution attainable from the various excitation transitions as labeled arising from the ^6^H_15/2_ ground state.^38^ The visible region encompassing the ^6^H_15/2_ → ^4^I_15/2_ transition around 450 nm is particularly attractive for white light-emitting applications by use of a blue light-emitting diode (LED) as the excitation source.^51^ It is observed in Figure 8 that while the host shows mere baseline behavior, the peak intensities increase from the 05Dy to the 1Dy glass and thereafter diminish progressively for the 2–4Dy glasses. The concentration dependence of the PL was further verified by exciting the prominent ^6^H_15/2_ → ^6^P_7/2_ transition at 350 nm leading to the emission spectra shown in Figure 9a. The various Dy^3+^ emissions assigned to the ^4^F_9/2_ → ^6^H_15/2_, ^4^F_9/2_ → ^6^H_13/2_, and ^4^F_9/2_ → ^6^H_11/2_ transitions^2,10,38^ are clearly seen for the 05–4Dy glasses. Here again, the emission is clearly most intense for the 1Dy glass and weakens for the 2–4Dy glasses. The inset in Figure 9a shows a plot of the three-band integrated PL intensities as a function of Dy_2_O_3_ concentration for a graphical appraisal. The evolution of the intensities clearly contrasts with the absorption (Figure 7) which increased steadily with the Dy_2_O_3_ concentration. Herein, the data for the 1–4Dy glasses exhibiting a decreasing trend were subjected to linear regression yielding the correlation coefficient and equation displayed in the inset of Figure 9a. The situation resembles the case of Nd^3+^ ions in the barium glass system wherein the maximum PL was realized for 1 mol % Nd_2_O_3_.^17^ Thus, as it was the case with Nd^3+^ ions,^17^ even though some depolymerization of the glass network was induced by Dy^3+^ (*vide supra*) which facilitates solubilizing the cations, the phenomenon of concentration quenching ensued at comparably elevated Dy^3+^ concentrations. A strong Dy^3+^–Dy^3+^ distance dependence of the interactions leading to quenching is then suggested for interionic distances estimated at 11.91, 10.39, and 9.47 Å for the 2Dy, 3Dy, and 4Dy glasses, respectively (Table 2). The aspect of the interionic distance dependence of the quenching shall be brought back to discussion with decay curve analysis (*vide infra*).

As a parameter relevant to lighting applications, the intensity ratios of the yellow (^4^F_9/2_ → ^6^H_13/2_) to blue (^4^F_9/2_ → ^6^H_15/2_) emissions (*Y*/*B*)^8,12,20,44,50,52,53^ were obtained for the 05–4Dy glasses which are reported in Table 8. It is observed that the *Y*/*B* ratio is lowest for the 1Dy glass exhibiting the most intense overall emission in Figure 9a, whereas the highest value pertains to the 4Dy glass with weakest PL. The various Dy^3+^ spectra in Figure 9a were further characterized *via* the Commission Internationale de l’Eclairage (CIE) 1931 chromaticity diagram as shown in Figure 9b. The corresponding chromaticity coordinates (*x*,*y*) determined for the 05–4Dy glasses are also presented in Table 8. The values are in general similar to the reported for different Dy^3+^-doped phosphate glasses.^12,14,20^ Notably, the 1Dy glass is most shifted toward white light emission in harmony with the lowest *Y*/*B* ratio. By contrast, the 4Dy glass with the highest *Y*/*B* ratio has the coordinate shifted farthest toward the yellow. Further on, we proceed to calculate the correlated color temperature (CCT) based on the following equation^20,50,52,54^
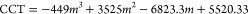
5where  and (*x*, *y*) are the chromaticity coordinates, calculated from the emission spectra. The different values obtained are presented in Table 8 with the other parameters. Overall, the CCT values are in the range of 4000–5000 K which is desirable for lighting applications.^12^ Most notably, the 1Dy glass shows the highest CCT at 4701 K consistent with the CIE coordinate shifted toward white light emission as shown in Figure 9b.

**Table 8:** Yellow to Blue Ratio (*Y*/*B*), CIE 1931 Chromaticity Coordinates (*x*,*y*), and Color Correlated Temperatures (CCT) Obtained for the Dy^3+^-Containing Glasses

glass	*Y*/*B*	*x*	*y*	CCT (K)
05Dy	1.68	0.364	0.414	4632
1Dy	1.58	0.361	0.411	4699
2Dy	1.63	0.362	0.413	4680
3Dy	1.76	0.372	0.422	4464
4Dy	1.78	0.372	0.423	4468

**Figure 10:**
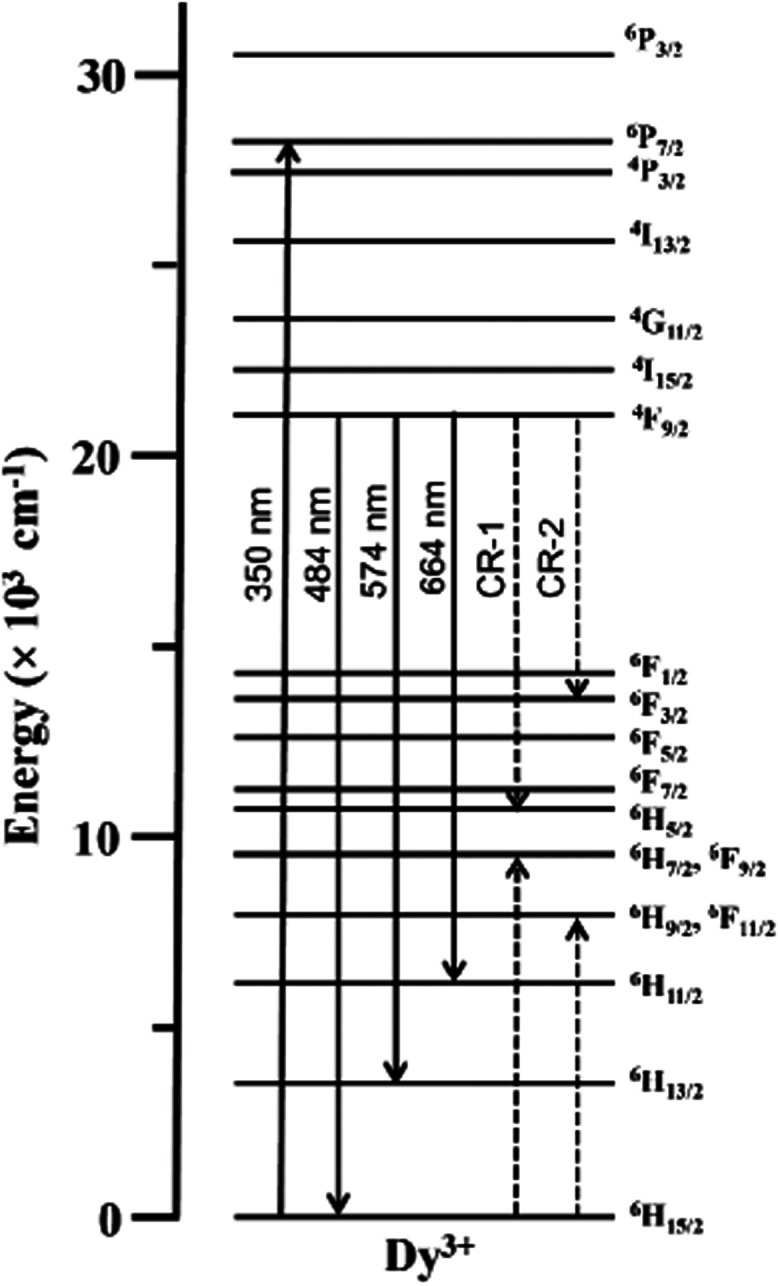
Partial energy level diagram illustrating ^6^H_15/2_ → ^6^P_7/2_ excitation in Dy^3+^ ions at 350 nm along with various ^4^F_9/2_ → ^6^H_15/2_, ^6^H_13/2_, and ^6^H_11/2_ emissions and the cross-relaxation channels CR-1: (^4^F_9/2_ + ^6^H_15/2_) → ^6^H_5/2_ + (^6^H_7/2_, ^6^F_9/2_) and CR-2: (^4^F_9/2_ + ^6^H_15/2_) → ^6^F_3/2_ + (^6^H_9/2_, ^6^F_11/2_).

Similar to the present work, the concentration quenching effect has been observed for different glasses at relatively high Dy^3+^ concentrations.^3,10−12,14,20,52^ For instance, Madhukar Reddy et al.^3^ analyzed 20PbO-5CaO-5ZnO-10NaF-(60 – *x*)-B_2_O_3_-*x*Dy_2_O_3_ glasses where *x* = 0.1, 0.25, 0.5, 1.0, and 2.0 mol % and observed the highest Dy^3+^ emission for 1.0 mol % Dy_2_O_3_. Shoaib et al.^10^ reported that the maximum PL of Dy^3+^-doped lithium–gadolinium phosphate glasses was highest for 1 mol % Dy_2_O_3_ with subsequently quenched emission. In their study of Dy^3+^-doped potassium–zinc–calcium phosphate glasses, Mahamuda et al.^11^ likewise observed maximum emission for 1 mol % Dy_2_O_3_. Maheshwari et al.^12^ evaluated Dy^3+^-doped barium–zinc–lithium phosphate glasses and reported the optimum output for 1.5 mol % Dy_2_O_3_. Jlassi et al.^14^ reported similar emission performance of zinc lead fluoride sodium phosphate glass having 1.0–1.5 mol % Dy_2_O_3_ but quenching at 2.0 mol %. The work of Shwetha and Eraiah^20^ on lithium zinc phosphate glasses was then consistent with an optimum PL with 1 mol % Dy_2_O_3_ followed by concentration quenching. The study conducted by Farooq et al.^52^ on dysprosium-doped antimony–magnesium–strontium-oxyfluoroborate glasses showed the maximum emission for 0.5 mol % Dy_2_O_3_ being close to 1.0 mol % Dy_2_O_3_ and then followed by clear quenching at higher Dy_2_O_3_ concentrations. The present results, e.g., Figure 9a, then show consistency with various reports on Dy^3+^ PL and the concentration quenching effect in various glasses, in particular phosphate based. The energy difference of the lowest transition from ^4^F_9/2_ to ^6^H_11/2_ is herein estimated at about 15060 cm^–1^ and this value is much higher than the average phonon energy in phosphate glasses of about 1200 cm^–1^.^15^ This inhibits electron–phonon coupling and thereby multiphonon relaxation for concentration quenching of Dy^3+^ ions may be ruled out. Hence, the typical nonradiative processes considered at the origin of the PL weakening in Dy^3+^-doped glasses are various cross-relaxation (CR) channels involving nearby Dy^3+^ ions interacting^2,14,20,44,50,52^ and/or excitation migration *via* resonance energy transfer (RET).^10,12^ Some of the CR pathways commonly invoked are depicted in the simplified schematic in Figure 10 which are labeled CR-1: (^4^F_9/2_ + ^6^H_15/2_) → ^6^H_5/2_ + (^6^H_7/2_, ^6^F_9/2_) and CR-2: (^4^F_9/2_ + ^6^H_15/2_) → ^6^F_3/2_ + (^6^H_9/2_, ^6^F_11/2_).

**Figure 11:**
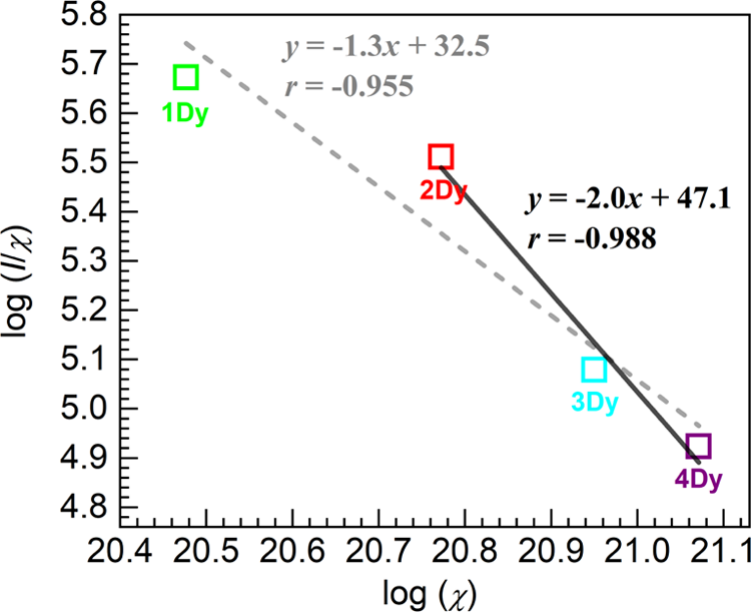
Plot of log(*I*/χ) vs. log(χ) for the glasses wherein PL quenching was manifested (*I* is the integrated PL intensity of the three transitions in Figure 9a; χ is the concentration of Dy^3+^ ions per cm^3^ from Table 2). The dashed and solid lines are linear fits to the data encompassing the 1–4Dy and 2–4Dy glasses, respectively (equations and correlation coefficients, *r*, displayed).

According to Dexter’s theory,^55,56^ the transfer rate, *T*, for nonradiative interactions between ions is given by the general expression
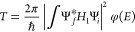
6where Ψ_*i*_ and Ψ_*f*_ are the initial (the energy donor is excited while the acceptor is relaxed) and final (the energy donor is relaxed while the acceptor is excited) states, respectively; φ(*E*) is the density of states provided by vibrational motion; and *H*_1_ is the Hamiltonian for the Coulombic interaction which upon expansion gives rise to the several terms associated with the physical mechanisms as electric dipole–dipole, dipole–quadrupole, quadrupole–quadrupole, electric dipole–magnetic dipole and exchange interactions. Among these, the leading term is that of the electric dipole–dipole interaction, which has also been considered at the origin of concentration quenching in Dy^3+^-doped glasses.^2,4,10,45,57,58^ In this context, a common approach to evaluate the type of multipolar interaction leading to the PL quenching is through the relationship among the integrated PL intensity (*I*) and the dopant concentration (χ) *via* the following equation^57,58^
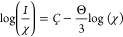
7where *Ç* is a constant and Θ relates to the type of interaction being equal to 6, 8, or 10 for the electric dipole–dipole, dipole–quadrupole, or quadrupole–quadrupole mechanisms, respectively. Such evaluation was therefore carried out here through a plot of log(*I*/χ) vs. log(χ) for the 1–4Dy glasses encompassing the range wherein the PL quenching was manifested as shown in Figure 11. A first attempt of linear regression for the entire data set yielded a correlation coefficient *r* = −0.955 and a slope of −1.3 leading to the Θ parameter being estimated at 3.9. Zhang et al.^57^ performed this type of analysis of their data and obtained a slope of −1.348 indicating that it supported the dipole–dipole mechanism. Furthermore, narrowing the range herein to the 2–4Dy glasses, which exhibited quenching, yielded an improved correlation coefficient *r* = −0.988 and remarkably a slope of −2.0 as shown in Figure 11. In this case, the value of Θ = 6.0 is achieved pointing clearly to the dominance of the dipole–dipole interaction underpinning the concentration quenching. The work of Xu et al.^58^ showed similar agreement reaching a value of Θ = 5.34 from the slope of the plot suggesting that the electric dipole interaction was the mode of energy transfer between Dy^3+^ ions. Various studies including analysis of decay curves performed through the Inokuti-Hirayama model have also agreed that the dipole–dipole interaction is the leading mechanism behind the PL quenching in Dy^3+^-doped glasses.^2,4,7,10,44,45,50,59^

The radiative properties such as transition probability, total radiative probability, branching ratio, stimulated emission cross-section, and radiative lifetime of the 1Dy glass may be calculated as the deciding parameters for applications such as gain medium in solid-state lighting technology.^37^ These important parameters were calculated with the help of computed intensity parameters by using the J–O theory. The radiative transition probability, *A*, of Dy^3+^ ions was calculated using the relation
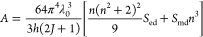
8where, λ_0_ is the emission peak wavelength, *n* is the refractive index of the 1Dy glass, and *S*_ed_ and *S*_md_ are the electric dipole (ed) and magnetic dipole (md) strengths, respectively, of the corresponding transition, Ψ_*J*_ → Ψ′_*J*′_, and are given by
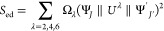
9
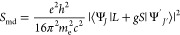
10The total radiative transition probability, *A*_T_, and radiative lifetime, τ_R_, of the metastable state of the Dy^3+^-doped glass were then determined using the following relations:
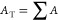
11
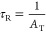
12The possibility of lasing emission was evaluated by the branching ratio of each emission transition as
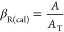
13The stimulated emission cross-section of observed emission bands, σ_se_, were determined by
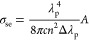
14where, λ*p* and Δλ*p* are the emission wavelength and effective bandwidth, respectively. The parameters for the different radiative properties determined for the 1Dy glass are given in Table 9. The hypersensitive electronic-dipole transition of the 1Dy glass at 574 nm owned optimum radiative parameters. The branching ratio of any emission transition more than 0.5 is considered suitable for lasing action (performance) and in the present case, it was found to be 0.71 for the 574 nm emission band. The stimulated emission cross-section compared to other transitions, ^4^F_9/2_→ ^6^H_13/2_ (39.2 × 10^–22^ cm^2^) were the highest for hypersensitive transition and this value comparable to other phosphate and tellurite glass matrices^11,12,44,45,47^ suggesting required population-inversion readily achievable for high-power yellow light lasing performances. Considering the radiative values, the ^4^F_9/2_ → ^6^H_13/2_ emission transition is suitable for solid-state yellow light applications under high-power near-UV LED excitation.

**Table 9:** Emission Peak Wavelength (λ_p_, nm), Effective Bandwidth (Δλ_p_, nm), Radiative Transition Probabilities (*A*, s^–1^), Total Radiative Transition Probabilities (*A*_T_, s^–1^), Stimulated Emission Cross-Section (σ_se_ × 10^–22^ cm^2^), Experimental and Branching Ratios (β_R(cal)_), Radiative Lifetime (μs) of the 1Dy Glass (Present Work) and Comparison with Reported Values for Other Dy^3+^-Doped Glasses

transition ^4^F_9/2_ →	parameter	1Dy	PDy10^11^	PDy1^12^	NPZDy1.0^14^	BPAPbLiDy1^44^	TYDF1^45^	TZONBSFDy0.5^47^
^6^H_15/2_	λ_p_	481	483	483	482	478	480	481
	Δλ_p_	15.7	12.9	12.3	17.08	8.83	21.86	16.88
	*A*	280	266	109	994	95	—	133
	σ_se_	4.9	3.49	2.51	1.76	2.88	3.05	1.54
	β_R(cal)_	0.22	0.14	0.11	0.43	0.20	—	0.15
^6^H_13/2_	λ_p_	574	573	575	576	572	575	576
	Δλ_p_	13.64	10.6	13.22	13.62	6.117	17.38	15.48
	*A*	918	1446	824	1254	330.8	—	666.2
	σ_se_	39.2	45.6	35.32	5.68	29.27	32.2	15.42
	β_R(cal)_	0.71	0.74	0.87	0.54	0.70	—	0.76
^6^H_11/2_	λ_p_	663	663	663	665	658	659	663
	Δλ_p_	15.64	25	14.53	17.34	6.66	25.11	14.65
	*A*	100	249	16.6	78	44.78	—	79.74
	σ_se_	5.8	3.34	1.13	0.49	1.4	4.47	2.84
	β_R(cal)_	0.08	0.13	0.02	0.03	0.10	—	0.09
	*A*_T_	1298	1961	949.6	2326	470.8	—	878.94
	τ_R_	770	509	1005	430	2100	—	1130

**Figure 12:**
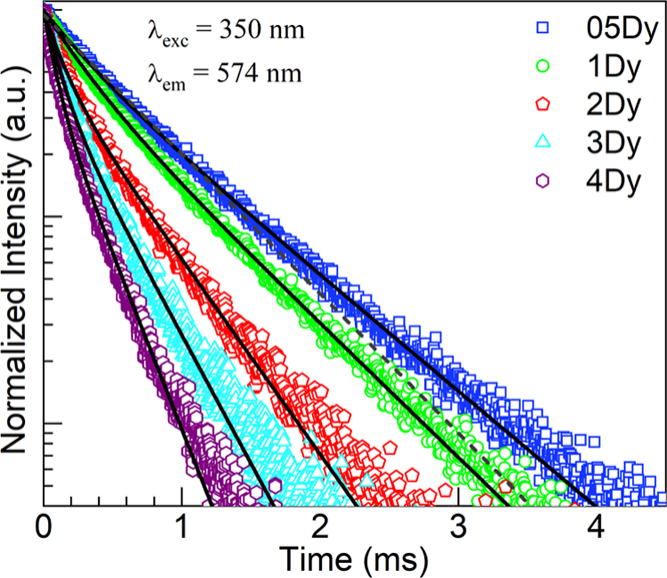
Semilog plots of normalized emission decay curves obtained for the 05–4Dy glasses under ^6^H_15/2_ → ^6^P_7/2_ excitation at 350 nm by monitoring ^4^F_9/2_ → ^6^H_13/2_ emission at 574 nm. The solid traces are biexponential fits (deduced parameters in Table 10) to the data for the various glasses; the dashed trace is the single-exponential fit attempted for the 05Dy glass.

We turned our attention at this point to examine the emission decay kinetics of Dy^3+^ ions in 05–4Dy glasses. The emission decay curves were recorded under ^6^H_15/2_ → ^6^P_7/2_ excitation at 350 nm by monitoring the ^4^F_9/2_ → ^6^H_13/2_ emission at 574 nm. These are shown in the semilog plots in Figure 12, which were normalized for comparison. The decay curves show nonexponential behavior and noticeably appear to decay faster with increasing Dy_2_O_3_ concentration. This type of behavior is characteristic of Dy^3+^-doped glasses and reflects strong ion–ion interactions with increasing Dy^3+^ concentrations.^2,4,7,10,14,46,60^ The normalized decay curves were then fit to a biexponential function as customary^11,14,46,52,60^
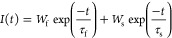
15where *I*(*t*) is the time-dependent luminescence intensity, *W*_f_ and *W*_s_ are pre-exponential weight factors, and τ_f_ and τ_s_ are fast and slow decay times, respectively. The solid traces in Figure 12 represent the fits, and the parameters deduced are presented in Table 10 along with the estimated errors. The τ_f_ and τ_s_ decay components deduced are deemed linked to two Dy^3+^ ion populations; namely, the fast lifetime can be attributed to the decay of strongly interacting Dy^3+^ ions (e.g., through CR-1 and CR-2 channels, Figure 10) whereas the slow lifetime relates to the decay of Dy^3+^ ions lacking the strong influence from the Dy^3+^–Dy^3+^ transfer.^2^ The values are all seen to decrease in going from the 05Dy to the 4Dy glass even though the PL was not quenched for the 1Dy glass, which exhibited the maximum emission. A single-exponential fit was attempted on the 05Dy glass with the lowest Dy^3+^ concentration as shown in the overlay in Figure 12 (dashed trace) yielding a lifetime of 644.4 (±0.5) μs. However, it was noticed that the fit was not as suitable for matching the experimental data as the biexponential function. This further supports that the cross-relaxation pathways still manifest at low Dy^3+^ concentrations, while the PL can still improve. Herein, an estimate of the population of Dy^3+^ ions with slow decay time, *P*_s_, can be obtained from the parameters deduced using the following equation^2,60^
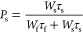
16The estimated percentages for the 05Dy, 1Dy, 2Dy, 3Dy, and 4Dy glasses are 85.3, 79.2, 81.4, 74.3, and 74.1%, respectively. Hence, the percentages of Dy^3+^ ions with fast decay time, *P*_f_, are 14.7, 20.8, 18.7, 25.7, and 25.9%, for the 05Dy, 1Dy, 2Dy, 3Dy, and 4Dy glasses correspondingly. Although some fluctuation is observed, overall, there seems to be a tendency for the population of interacting Dy^3+^ ions to increase with Dy_2_O_3_ content. This suggests that the increase in Dy^3+^ concentration leading to shorter Dy^3+^–Dy^3+^ distances promotes the involvement in the energy transfer processes that increase the nonradiative decay rates such as the CR-1 and CR-2 pathways (Figure 10). In addition, an average lifetime, τ_ave_, may be calculated for each glass from the following equation^11,14,44,52^
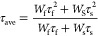
17The average lifetimes obtained also decreased consistently and were found to be 706, 587, 399, 288, and 210 μs for the 05Dy, 1Dy, 2Dy, 3Dy, and 4Dy glasses, respectively. The experimental lifetime average value of the 1Dy glass may be brought to comparison with the radiative decay rate calculated (Table 9) to estimate the quantum efficiency, η, from the following equation^7,11^
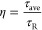
18The resulting quantum efficiency is 76.2% which is comparable to the reported for different Dy^3+^-doped phosphate glasses.^7,11,12,14,44^

**Table 10:** Fast (τ_f_) and Slow (τ_s_) Decay Times of Dy^3+^ Ions along with Corresponding Weight Factors (*W*_f_, *W*_s_) and Average Lifetimes Estimated for the 05-4Dy Glasses by Fitting the Experimental Decay Curves in Figure 12 with a Biexponential Decay Function

glass	*W*_f_	τ_f_ (μs)	*W*_s_	τ_s_ (μs)
05Dy	0.301 (±0.005)	305.5 (±3.5)	0.689 (±0.005)	774.7 (±2.5)
1Dy	0.397 (±0.003)	266.1 (±1.6)	0.599 (±0.004)	671.3 (±1.7)
2Dy	0.442 (±0.003)	129.5 (±0.9)	0.541 (±0.003)	461.4 (±1.3)
3Dy	0.526 (±0.004)	106.9 (±0.7)	0.464 (±0.004)	350.2 (±1.5)
4Dy	0.530 (±0.002)	78.3 (±0.4)	0.463 (±0.003)	256.3 (±0.8)

**Figure 13:**
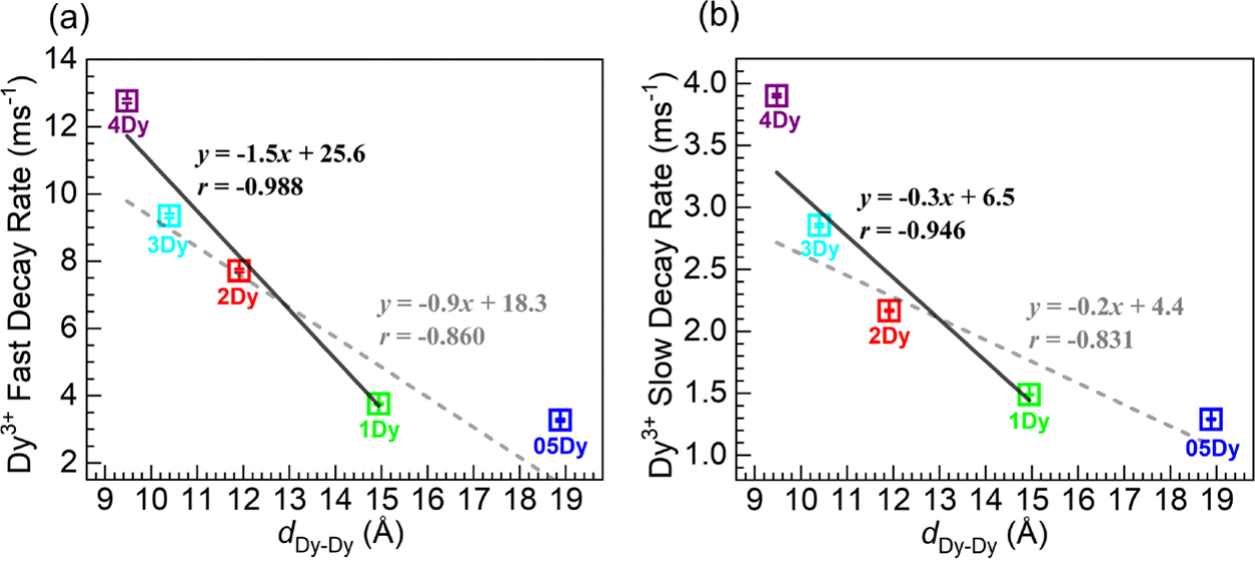
Plots of (a) fast and (b) slow ^4^F_9/2_ emission decay rates of Dy^3+^ ions as a function of the mean Dy^3+^–Dy^3+^ distances, *d*_Dy–Dy_ (values listed in Table 2) in the glasses; the symbols are color coded and the glass codes placed underneath. The dashed and solid lines are linear fits to the data encompassing the 05–4Dy and 1–4Dy glasses, respectively (equations and correlation coefficients, *r*, displayed).

The continuous shortening of Dy^3+^ lifetimes with increasing Dy^3+^ concentration being manifested even when the PL increases as observed (e.g., Figure 9a, Table 10) has been similarly reported for a variety of Dy^3+^-doped glasses.^3,10−12,14,44−47^ Nonetheless, despite this being the standard outcome regarding the Dy^3+4^F_9/2_ excited-state lifetime, this phenomenon is not well understood. Interestingly, analogous emission decay trends have been observed for Nd^3+^ ions in the barium phosphate glass system^17^ which concurred with reports from other groups on neodymium laser glasses.^61,62^ Based on the analysis performed, it was suggested that the Nd^3+^ concentration quenching took place primarily via the excitation migration or “hopping” mechanism, whereas the lifetime decrease that was accompanied by a PL increase reflected the contribution of a cross-relaxation pathway.^17^ Herein, we propose an approach seeking new insights by evaluating the fast and slow Dy^3+4^F_9/2_ emission decay rates (*k*_Dy_ = τ ^–1^; τ values in Table 10) as a function of the interionic distances (*d*_Dy–Dy_, Table 2) through the plots shown in Figure 13a,b. In an initial assessment, the data for both fast and slow decay rates were fit by linear regressions for all Dy^3+^-containing glasses (dashed traces) yielding correlation coefficients *r* of −0.860 and −0.831, respectively, denoting weak correlations for the entire concentration range. On the other hand, as shown in Figure 13a,b, a second attempt was made to fit the data for the 1–4Dy glasses (solid traces) given that the emission was highest for the 1Dy glass and was quenched thereafter [e.g., Figure 9a]. In this case, the correlation coefficients were noticeably higher at −0.988 and −0.946, for the fast and slow decay rates, respectively. This implies a stronger correlation when the Dy^3+^–Dy^3+^ mean distances were within the 14.94–9.47 Å range (Table 2). Besides, we may also consider that the decay curve fittings in Figure 12 (*vide supra*) indicated that even for the 05Dy glass the cross-relaxation channels (e.g., CR-1 and CR-2) were operating. Thereafter, the PL output of the 1Dy glass improved while the decay times decreased. Consequently, this suggests that the presence of energy transfer via cross-relaxation channels at low concentrations does not prevent emission enhancement. This type of transfer thus seems to have less impact on the Dy^3+^ emission intensity at low concentrations as it was similarly suggested for low concentrations of Nd^3+^ ions in the glass system.^17^ Conversely, the concentration quenching ensues at higher concentrations in the 2–4Dy glasses and may then proceed prominently via the resonant transfer or excitation migration mechanism in addition. This is supported by the fact that the slow lifetimes assumed to not be impacted by the cross-relaxation channels decreased continuously (Table 10) while their population remained relatively high for the 2Dy, 3Dy, and 4Dy glasses at 81.4, 74.3, and 74.1%, respectively. However, the fact that the correlation appeared to be stronger for the faster decay rates in Figure 13 suggests the key involvement of the interacting ions through the cross-relaxation pathways. Connecting this analysis with the results of the plot in Figure 11, it is inferred that the transfer behind the efficient quenching occurs through the electric dipole–dipole mode, which becomes clearly manifest at high Dy^3+^ concentrations.

## 4 Summary and Conclusions

In brief, Dy^3+^-doped phosphate glasses of interest to light-emitting devices were prepared by melting with 50P_2_O_5_-(50 – *x*)BaO-*x*Dy_2_O_3_ (0 ≤ *x* ≤ 4 mol %) nominal compositions and studied comprehensively through various measurements including refractive index, density, XRD, Raman spectroscopy, X-ray photoelectron spectroscopy, dilatometry, optical absorption, and PL spectroscopy with decay kinetics assessment. The refractive index and densities were in general found to increase with Dy_2_O_3_ content up to values of 1.6120 and 3.823 g/cm^3^, respectively, for glass with 4 mol % Dy_2_O_3_. The concentration of Dy^3+^ ions then increased in the 1.495–11.78 × 10^20^ ions/cm^3^ range indicating that Dy^3+^–Dy^3+^ mean distances decreased within the 18.88–9.47 Å range. The structural analyses indicated that a slight depolymerization of the phosphate network was induced by high concentrations of Dy^3+^ ions replacing Ba^2+^. The thermal analysis showed a tendency for the glass transition and softening temperatures to increase, while the thermal expansion coefficient decreased with the Dy_2_O_3_ content. The results indicating a glass strengthening effect accompanied by increased glass rigidities were interpreted in terms of the increasing concentration of Dy^3+^ ions with high-field strength (*F*_Dy(III)_*=* 3.607 Å^–2^) replacing Ba^2+^ ions with lower field strength (*F*_Ba(II)_ = 0.992 Å^–2^). In this context, a comparison was made with the currently examined glass with 4 mol % Dy_2_O_3_ and the reported thermal expansion data for glasses containing 4 mol % of Nd_2_O_3_, Gd_2_O_3_, Yb_2_O_3_,^19^ and Eu_2_O_3_,^16^ which supported the high ionic field strengths of the lanthanides driving the changes in thermal expansion coefficients.

Concerning the optical properties, the Dy^3+^ optical absorption increased linearly with Dy_2_O_3_ concentration, supporting the effective inclusion of Dy^3+^ ions, likely assisted by network depolymerization. However, the PL intensity was the highest for 1 mol % Dy_2_O_3_ and decreased for higher concentrations. Hence, Judd–Ofelt and radiative property calculations were carried out for such a glass. The Ω_2_ > Ω_4_ > Ω_6_ trend in J–O parameters indicated that the Dy^3+^ ions are positioned in more asymmetric sites and connected with stronger covalent bonds with surrounding oxygen ligands. As an important outcome of the radiative parameters, the ^4^F_9/2_ → ^6^H_13/2_ emission transition around 574 nm for the glass with 1 mol % Dy_2_O_3_ had a branching ratio at 0.71 deemed suitable for solid-state yellow lasing applications under high-power near-UV LED excitation. Further PL evaluations were carried out including CIE 1931 color coordinates and correlated color temperatures pointing to the potential for lighting applications of the 1 mol % Dy_2_O_3_-doped glass given its shifted coordinate toward white light and correlated color temperature of 4701 K. A detailed analysis of the PL spectra in the context of Dexter’s theory supported that the interaction leading to quenching at high Dy_2_O_3_ contents is of the electric dipole–dipole type. Further analysis of the emission decay curves suggested that the cross-relaxation channels between Dy^3+^ ions taking place at low concentrations are responsible for the lifetime-shortening effect that takes place while the PL increases. Contrarywise, high Dy^3+^ concentrations facilitate the emission quenching likely including additionally the resonant excitation migration pathway for Dy^3+^–Dy^3+^ mean distances shorter than ∼15 Å. The present results provide important physicochemical insights as well as various parameters of practical value, which may become useful for developing Dy^3+^-doped glasses for solid-state lighting and lasing applications.
